# A27 DNA HYPOMETHYLATION INDUCED BY 5-AZA-CDR OR LOSS OF DNMT1 INHIBITS COLITIS-ASSOCIATED COLORECTAL CANCER

**DOI:** 10.1093/jcag/gwac036.027

**Published:** 2023-03-07

**Authors:** F Larsen, H Good, A Shin, M Derouet, L Zhang, S Asfaha

**Affiliations:** Department of Medicine, Western University, London, Canada

## Abstract

**Background:**

Colorectal cancer is the second leading cause of cancer death in Canada. A major risk factor for the development of colorectal cancer is chronic inflammation leading to colitis-associated cancer (CAC). We previously described a CAC mouse model in which tumors arise from DCLK1+ tuft cells following loss of the tumor suppressor adenomatous polyposis coli (APC) and induction of colitis. Interestingly, both colitis and CAC display epigenetic changes that modulate gene expression. However, the impact of DNA methylation changes on colonic tumorigenesis is not known. Thus, we hypothesize that inhibition of DNA methylation in DCLK1+ tuft cells reduces colonic tumorigenesis.

**Purpose:**

In this study, we aim to investigate the role of DNA methylation in CAC by inhibiting DNA methylation using genetic and pharmacologic means.

**Method:**

Using a publicly available dataset (GSE75214) of gene expression data analyzed by microarray from colonic biopsies of patients with ulcerative colitis and Crohn’s Disease with active disease, we examined the expression of DNA methyltransferases (DNMTs). Expression of DNMTs in mice with colitis was additionally examined by RT-qPCR and global DNA methylation levels measured by 5-mC ELISA. In separate experiments, Dclk1-CreERT2/Apc^f/f^ mice were crossed to DNMT1^f/f^ mice to knock-out the DNA methyltransferase DNMT1 in DCLK1+ tuft cells. Dclk1/Apc^f/f^ and Dclk1/Apc^f/f^/DNMT1^f/f^ mice were then administered three doses of tamoxifen followed by 2.5% dextran sodium sulfate (DSS) for five days to induce colitis. Fourteen weeks later, we assessed colonic tumor number and size. In a separate cohort of Dclk1/Apc^f/f^ mice, we induced colitis and treated the mice with six doses of the DNA de-methylating drug 5-AZA-2’-deoxycytidine (5-AZA) or vehicle, and assessed colonic tumor number. To examine DNA methylation changes, we then treated WT mice with 5-AZA and DSS and isolated intestinal epithelial cells. From the intestinal epithelial cell, we isolated DNA and ran the Infinium MouseMethylation BeadChip Array.

**Result(s):**

Patients with IBD were found to have increased expression of DNMT1 compared to healthy controls. Mice treated with DSS similarly had increased DNMT1 expression, as well as, global methylation levels compared to controls. Deletion of DNMT1 in DCLK1+ cells significantly inhibited the number and size of colonic tumors. Treatment of mice with 5-AZA decreased global and gene specific DNA methylation levels, and significantly reduced both the number of mice with tumors, and the average colonic tumor number and size per mouse.

**Conclusion(s):**

Our findings demonstrate that colitis in both patients and mice is associated with DNA methylation. Furthermore, DNA hypomethylation by 5-AZA treatment or loss of DNMT1 reduces CAC formation suggesting that altered DNA methylation plays a critical role in colonic tumorigenesis.

**Disclosure of Interest:**

None Declared

